# Flaky FeSiAl alloy-carbon nanotube composite with tunable electromagnetic properties for microwave absorption

**DOI:** 10.1038/srep35377

**Published:** 2016-10-20

**Authors:** Lina Huang, Xiaofang Liu, Dan Chuai, Yaxin Chen, Ronghai Yu

**Affiliations:** 1 School of Materials Science and Engineering, Beihang University, Beijing 100191, China

## Abstract

Flaky FeSiAl alloy/multi-wall carbon nanotube (FeSiAl/MWCNT) composite was fabricated by facile and scalable ball milling method. The morphology and electromagnetic properties of the FeSiAl alloy can be well tuned by controlling the milling time. It is found that the magnetic loss of the FeSiAl alloy is improved by optimizing the milling time due to the increased anisotropy field. Meanwhile the addition of MWCNTs enhances the dielectric loss of the composite by increasing the interfacial polarizations, dipolar polarizations and conductive paths. Relative to conventional FeSiAl absorbers, the FeSiAl/MWCNT composite exhibits greatly improved microwave absorption performance with advantages of strong absorption and small thickness. The minimum reflection loss of the composite reaches −42.8 dB at 12.3 GHz at a very thin thickness of 1.9 mm.

The emerging hazards of electromagnetic (EM) radiation on human health and electrical equipments, which derive from the applications of wireless communications and the widespread use of microwave devices, have become a serious environmental pollution problem and drawn much attention all over the world^1^,^2^,^3^. Microwave absorbing materials offer an effective method to solve this issue by converting EM energy to thermal energy or dissipating it by interference^4^. With the drastically increasing utilization of EM wave in gigahertz range, magnetic metals and alloys have recently received considerable attention among various microwave absorbing candidates because of their attractive advantages of high permeability and compatible permittivity. Until now, many traditional magnetic materials, such as Fe, Ni, Co, their alloys and compounds with size from nanometer scale to micrometer scale, have been widely investigated^5^,^6^,^7^,^8^,^9^,^10^,^11^. However, these magnetic absorbers often suffer from sharp decrease of permeability in gigahertz frequency due to the Snoek’s limit^12^, large matching thickness and large weight, which reduce their performance and limit their practical applications. To overcome the Snoek’s limit, a few efforts have been made to synthesize magnetic materials with large shape anisotropy. For example, Feng *et al.* improved the microwave absorbing performance of granular-shaped FeNi alloy by flattening them into flake-shaped FeNi alloy via mechanical alloying^13^. Tong *et al.* synthesized urchin-like Fe_3_O_4_ nanostructures and obtained minimum reflection loss (RL) of −29.96 dB at a thickness of 4.0 mm^14^.

To further improve the comprehensive performance of magnetic absorber, carbonaceous materials are usually used as dielectric addictive to construct heterogeneous composite absorber. Typically, CNTs are considered as good candidates because of their high dielectric loss, low density and low cost^15^,^16^,^17^. The composite containing CNTs and magnetic materials possess both strong dielectric loss and magnetic loss. The good impedance matching property can be achieved by tuning the ratio of magnetic components and CNTs. Besides, the CNTs with one dimensional tubular structure can readily form interconnected network which provides additional conductive paths in composite, and thus benefit the microwave attenuation. So far, the improved microwave absorbing properties have been obtained in many magnetic materials/CNTs composites. For example, Che *et al.* synthesized α-Fe-filled CNT/epoxy composites which exhibited minimum RL of −25 dB at 11 GHz with thin thickness of 1.2 mm^18^. Zou *et al.* prepared Ni nano-wire filled multi-walled carbon nanotubes (MWCNTs) by CVD method. The Ni nano-wires/MWCNTs composite achieved the minimum RL value of −23.1 dB at 8.0 GHz with a thickness of 4.0 mm^19^. Yang *et al.* fabricated FeNi decorated CNTs composite, and obtained optimal RL of −15.4 dB at 16.5 GHz with thickness of 1.6 mm^20^. Nevertheless, the fabrication of most CNT-magnetic metal hybrids involves complex processes, severe synthetic conditions and high costs, which impose restrictions on their practical applications.

FeSiAl alloy is an important soft magnetic material with extensive applications, and it has been proven to exhibit potential microwave absorbing properties in gigahertz frequency region. However, it is still a big challenge for FeSiAl absorber to fulfill the requirements for high-performance microwave absorbing materials with strong absorption, broad absorption bandwidth, small thickness and light weight^21^,^22^,^23^,^24^,^25^,^26^,^27^,^28^. Based on the abovementioned approaches for improving magnetic absorber, the combination of FeSiAl with CNTs would be a promising method to achieve this goal. Moreover, to facilitate practical application, it is more desirable to develop a preparation method for FeSiAl composite absorber which is simple, low cost and appropriate for large-scale production.

In this work, we fabricated FeSiAl/MWCNT composite absorber by a simple, low-cost and scalable ball milling method. The EM parameters of the composite absorber were well controlled by adjusting the morphology, especially the aspect ratio (length/thickness) of the FeSiAl particles, and the addition content of MWCNTs. Excellent microwave absorbing performance with RL of −42.8 dB at a very thin thickness of 1.9 mm was achieved in the composite consisting of flaky FeSiAl and moderate amount of MWCNTs (2 wt.%). The possible absorbing mechanisms related with the special morphology of FeSiAl alloy and the synergetic effect of MWCNTs and alloy were discussed in detail.

## Experimental

### Preparation of flaky FeSiAl alloy

Commercial FeSiAl alloy powder was used as a beginning material in the current study. The raw powder was ball milled in a planetary mill with the ball-to-powder weight ratio of 5:1 at a rotation rate of 500 rpm. The milling operation was performed under argon protection at room temperature. The milling time was tuned from 4, 8 to 16 h. During the milling process, anhydrous ethanol was added as process control agent. Finally, the products were collected, washed and dried in an oven for 12 h at 60 °C.The samples milled for 4, 8 and 16 h were denoted as FeSiAl-4, FeSiAl-8 and FeSiAl-16.

### Preparation of FeSiAl alloy-MWCNT composites

The MWCNTs used in this experiment were purchased from DK nanotechnology Co. LTD. The FeSiAl alloy-MWCNT composites were obtained by ball milling the mixture of commercial FeSiAl powders and MWCNTs for 8 h. The mass fraction of MWCNTs in the mixture was 2 and 10 wt.%. Certain content of anhydrous ethanol was added into the mixture as the process control agent. Finally, the products were collected, washed and dried in an oven for 12 h at 60 °C.

### Characterization

The crystal structure of the samples was determined by X-ray diffraction (XRD) using a diffractometer equipped with Cu-*K*α source. The morphologies of the samples were observed using ZEISS Merlin scanning electron microscopy (SEM) and transmission electron microscopy (TEM, JEOL-2100F). The static magnetic properties of the products were measured at room temperature using a vibrating sample magnetometer (VSM).

The electromagnetic parameters were measured on a vector network analyzer (Agilent Technologies, PNA-L, N520C) in the frequency range of 2–18 GHz. Specimens used for the measurements were fabricated from a mixture containing 60 wt.% of paraffin and 40 wt.% of the as-prepared samples. And then the resulting absorber/paraffin mixtures were compressed into toroidal-shaped specimens with outer diameter of 7.00 mm and inner diameter of 3.04 mm.

## Results and Discussions

### Phase structure and morphology

**Figure 1:**
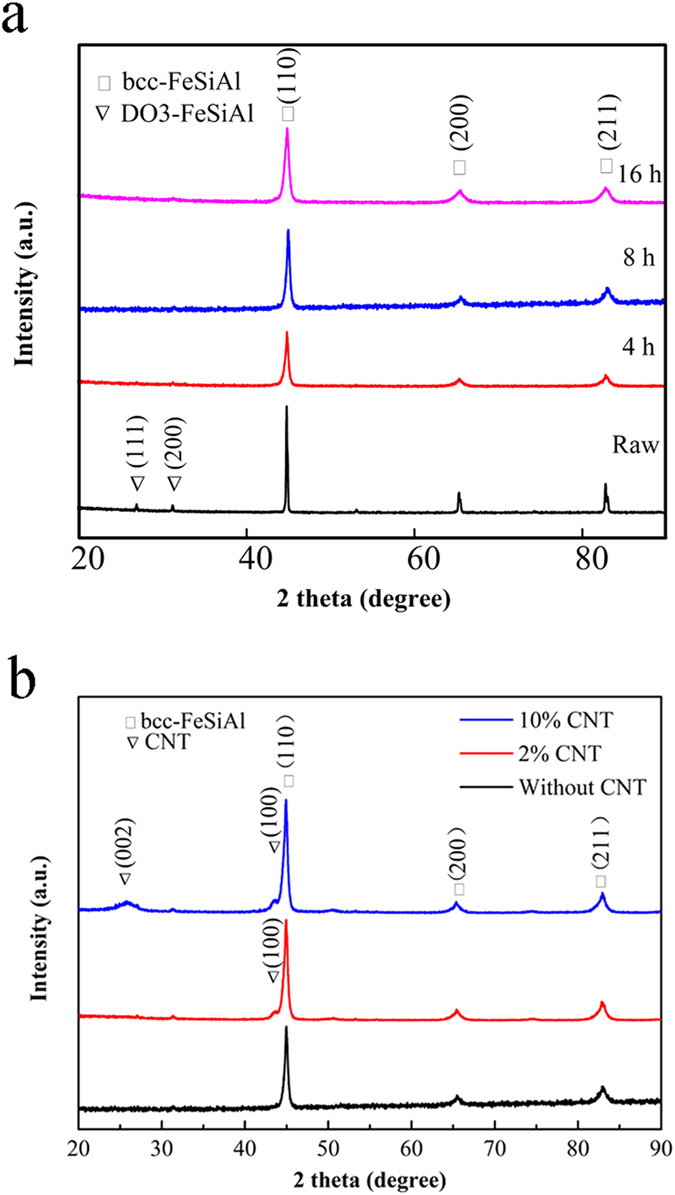
XRD patterns of (**a**) raw FeSiAl, FeSiAl-4, FeSiAl-8 and FeSiAl-16 samples, (**b**) FeSiAl-8 and FeSiAl/MWCNT composites.

Figure 1 shows the XRD patterns of the FeSiAl alloys milled for different time and the FeSiAl/MWCNTs composites with different MWCNT content. As shown in Fig. 1(a), the raw FeSiAl alloy consists of two phases. The diffraction peaks at 44.6°, 65.3° and 82.8° can be well indexed to the *α*-Fe (Si, Al) alloy with bcc structure, and the diffraction peaks at 27.0° and 31.0° originate from the DO3 structure. When the alloy was milled, the super lattice diffraction peaks of DO3 phase disappear. Meanwhile, the diffraction peaks from *α*-Fe (Si, Al) phase become broader and weaker, suggesting the decrease of FeSiAl particle size and the presence of internal strain induced by ball milling. According to Scherrer equation, the mean size of the raw FeSiAl alloy calculated using (110) peak is approximately 59.3 nm, while the mean sizes of the milled alloys decrease from 21.2, 19.4 to 16.1 nm as the milling time increases to 16 h. Figure 1(b) compares the XRD patterns of the FeSiAl-8 alloy and FeSiAl/MWCNT composites with different MWCNT content. It is clear that the addition of MWCNTs does not change the crystal structure of the FeSiAl alloy. When the MWCNT content is 10 wt.%, characteristic diffraction peaks at 26.3° and 43.4° from MWCNTs can be clearly detected^29^.

**Figure 2:**
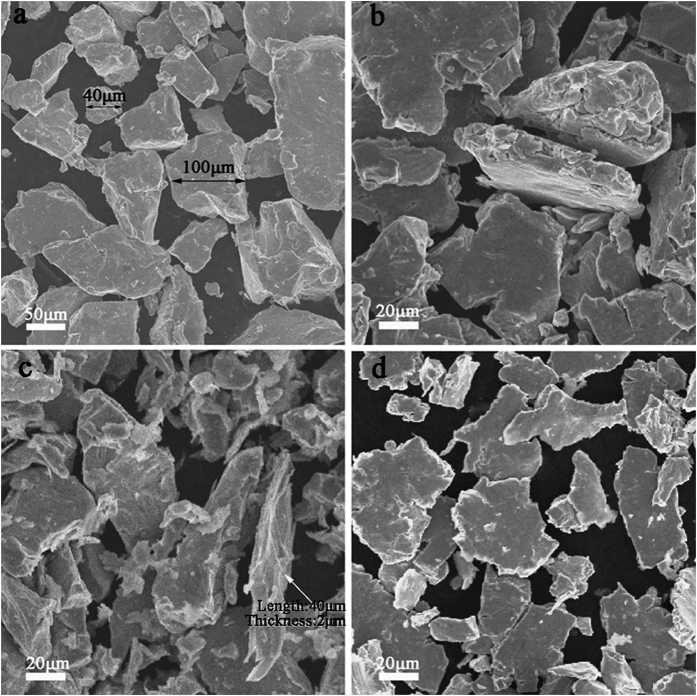
SEM images of (**a**) raw FeSiAl, (**b**) FeSiAl-4, (**c**) FeSiAl-8, and (**d**) FeSiAl-16 samples.

**Figure 3:**
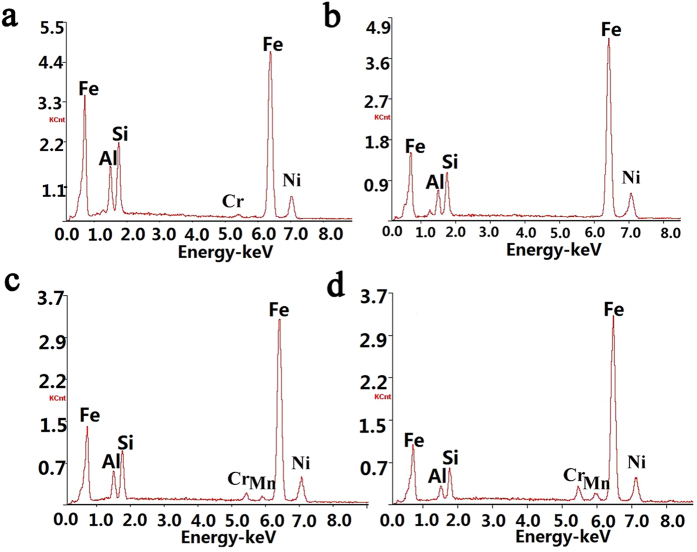
EDS results of (**a**) raw FeSiAl, (**b**) FeSiAl-4, (**c**) FeSiAl-8, and (**d**) FeSiAl-16 samples.

The typical morphologies of the raw and milled FeSiAl alloys are shown in Fig. 2. As observed in Fig. 2(a), the raw FeSiAl particles exhibit irregular morphology. After a short-time milling, there is no obvious morphology change for the FeSiAl-4 particles (Fig. 2(b)). When the milling time increases to 8 h, most particles appear to be flaky shape with large aspect ratio. As illustrated in Fig. 2(c), the representative FeSiAl sheet has a thickness of ~2 μm, a length of ~40 μm, and thus the corresponding aspect ratio (length/thickness) of the sheet is about 20:1. It is expected that the large aspect ratio of the FeSiAl sheets is favorable to increasing the magnetic loss in high-frequency region by overcoming the Snoek’s limit. Moreover, the thickness of the sheet is close to the typical skin depth of traditional FeSiAl alloy (~2 μm)^30^. which is also beneficial for suppressing the negative effect of the eddy current^31^,^32^,^33^ in alternating EM field. When the milling time was further elongated, more and more FeSiAl particles were crushed into fine pieces (Fig. 2(d)), which inversely decreased the aspect ratio of the sheets. Therefore, the morphology especially the aspect ratio of the FeSiAl particles can be well tuned through the ball milling process, which would significantly affect the microwave absorbing properties. In addition, the compositions of the FeSiAl alloys were measured by EDS. It reveals that the raw alloys are composed of Fe, Si, and Al elements with the atomic percentage of 74.9%, 13.9% and 11.2% respectively. Ball milling process has negligible effects on the composition of the alloys, as displayed in Fig. 3.

**Figure 4:**
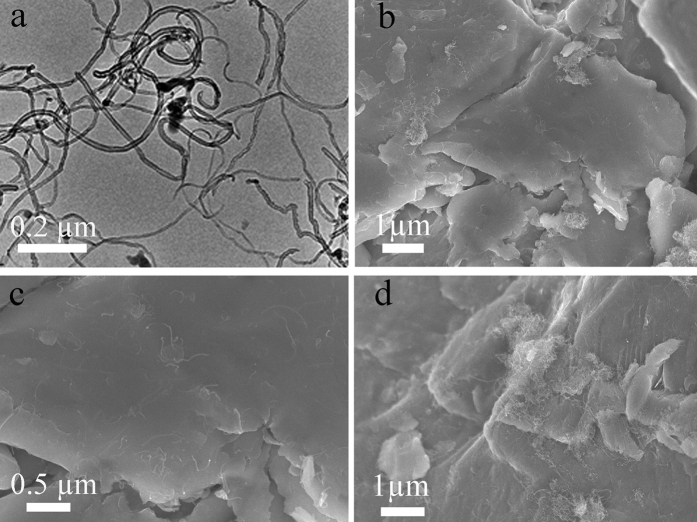
(**a**) TEM image of MWCNTs, (**b,c**) SEM images of FeSiAl/MWCNT composite (2 wt.% MWCNT), (**d**) SEM image of FeSiAl/MWCNT composite (10 wt.% MWCNT).

Figure 4 presents the typical morphologies of MWCNTs and FeSiAl/MWCNT composites milled for 8 h. It is found that the morphology of the FeSiAl sheets in composite is similar to that of the pure FeSiAl-8 sample. A large number of MWCNTs with diameter of 13–15 nm and length of several micrometers adhere to the surface of the FeSiAl sheets. Clearly, parts of the MWCNTs are well dispersed as shown in Fig. 4(c), while parts of MWCNTs are severely aggregated as observed in Fig. 4(d).

### Static magnetic properties

**Figure 5:**
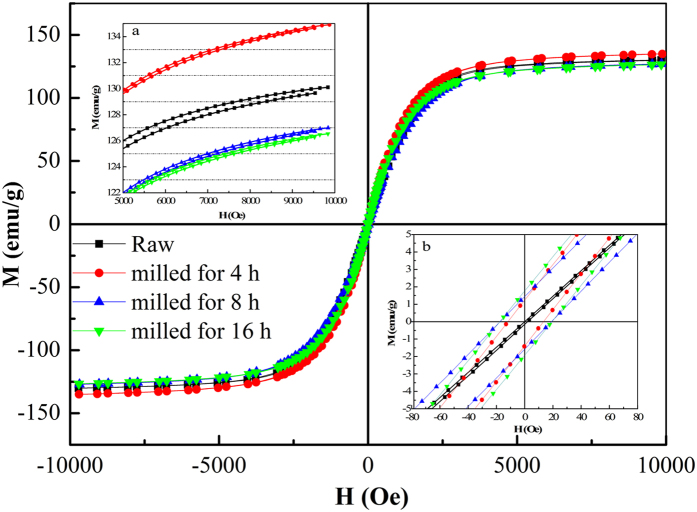
The hysteresis loops of raw FeSiAl, FeSiAl-4, FeSiAl-8 and FeSiAl-16 samples. The inset a is the enlargement of *M*_s_, and the inset b is the enlargement of hysteresis curves close to zero magnetic field.

The magnetization curves of the FeSiAl alloys were characterized by VSM at room temperature and presented in Fig. 5. All the samples exhibit typical soft magnetic characteristics with rather small remnant magnetization (*M*_*r*_) and coercivity (*H*_*c*_) values. Based on the measured data, the values of saturation magnetization (*M*_*s*_), *M*_*r*_ and *H*_*c*_ are summarized in Table.1. It is clear that ball milling has little effect on *M*_*s*_ while it significantly enhances *H*_*c*_ from 1.0 to 18.4 Oe as the milling time increases. The considerable increase of *H*_*c*_ is probably attributed to the formation of large amounts of structural defects and the presence of internal strain caused by the severe deformation, which may induce higher resistance to the magnetization because of the pinning effect^34^,^35^.

### Microwave absorbing performance

The FeSiAl alloys and FeSiAl/MWCNT composites were mixed with paraffin with filler loading of 40 wt.% for the measurements of EM parameters. Generally, the microwave absorbing performance of a material can be evaluated by the reflection loss (RL) value at a given thickness. According to transmission line theory, the RL of a normal incident EM wave can be calculated based on the measured EM parameters:


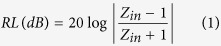



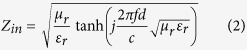


Where *Z*_*in*_ is the input impedance at the interface of free space and material, *f* is the frequency, *d* is the thickness of the absorber, *ε*_*r*_ and *μ*_*r*_ are the complex permittivity and permeability of the absorber, and *c* is the velocity of EM wave in free space.

**Figure 6:**
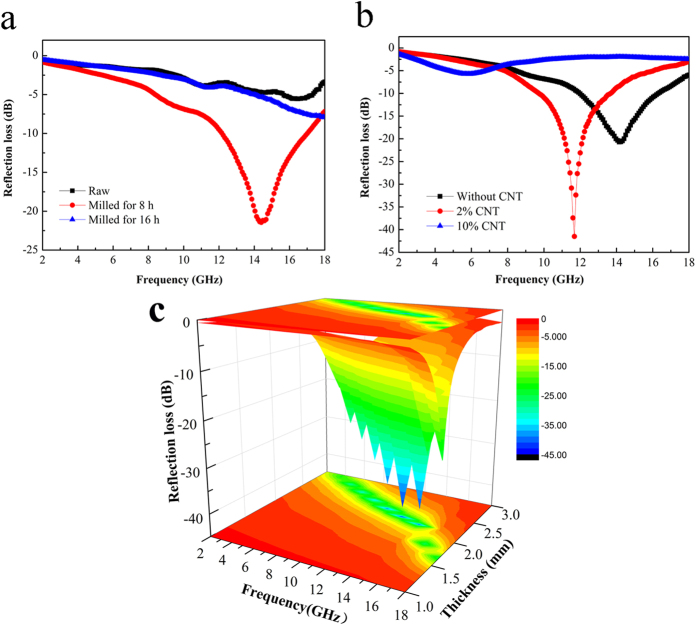
(**a**) Reflection losses of raw FeSiAl, FeSiAl-8, FeSiAl-16 samples with absorber thickness of 2 mm, (**b**) reflection losses of FeSiAl-8 and FeSiAl/MWCNT composites with absorber thickness of 2 mm, (**c**) 3D reflection loss plot of FeSiAl/MWCNT composite (2 wt.% MWCNT).

Figure 6 shows the calculated RL of the raw FeSiAl, milled FeSiAl alloys and FeSiAl/MWCNT composites in the range of 2–18 GHz with thickness of 2.0 mm. From Fig. 6(a), it is found that the milling time has important effects on the microwave absorbing performance of the FeSiAl alloys. Relative to the raw FeSiAl and FeSiAl-16 samples, the FeSiAl-8 sample with optimized milling time displays stronger microwave absorbing capability with minimum RL value of −21.4 dB at 14.4 GHz. The effective absorption bandwidth with RL less than −10 dB (corresponding to the 90% absorption of incident EM wave) of the FeSiAl-8 sample covers 4.9 GHz (12.1–17.0 GHz). For the purpose of further improving the microwave absorbing properties, a certain amount of MWCNTs were mixed with FeSiAl alloy and milled for 8 h. As shown in Fig. 6(b), the addition of MWCNTs with appropriate weight percentage of 2 wt.% results in the considerable enhancement of the microwave absorbing capability of the FeSiAl/MWCNT composite. To investigate the microwave absorbing performance in detail, three dimensional RL plot of the FeSiAl/MWCNT (2 wt.%) composite versus frequency in the range of 2–18 GHz and thickness in the range of 1.3–2.0 mm is presented in Fig. 6(c). It is found that the RL values of the composite can exceed −10 dB even with a very thin absorber thickness of 1.3 mm. The optimal RL value reaches −42.8 dB (at 12.3 GHz) with thickness of 1.9 mm, and the effective absorption bandwidth is up to 3.9 GHz (10.5–14.4 GHz). The microwave absorption properties of the FeSiAl/MWCNT (2 wt.%) composite together with other FeSiAl alloys and composites reported in recent literatures were summarized in Table 2. In comparison with other FeSiAl-based materials, the FeSiAl/MWCNT composite prepared in this work exhibits superior performance at a rather thin thickness, indicating the promising perspective of the FeSiAl/MWCNT absorber in the development of lightweight, thin microwave absorbing coatings.

**Figure 7:**
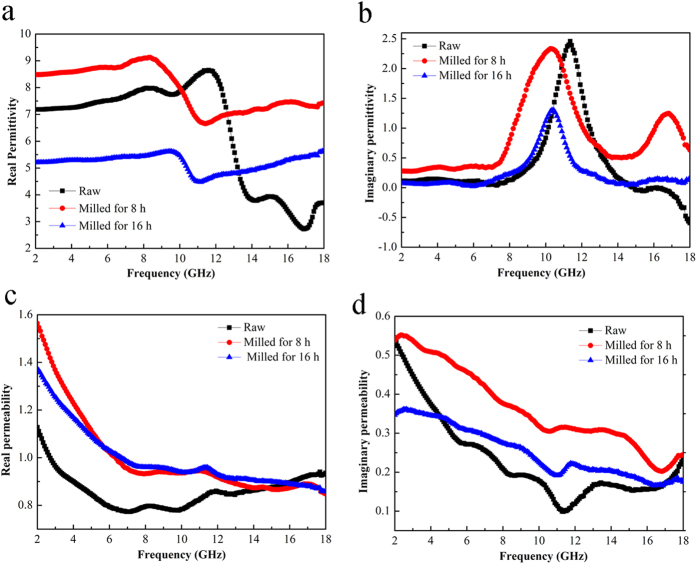
The frequency dependence of complex permittivity and permeability of raw FeSiAl, FeSiAl-8 and FeSiAl-16 samples.

To reveal the possible microwave absorbing mechanisms, the EM parameters of the FeSiAl alloys and FeSiAl/MWCNT composites are discussed in detail. The excellent microwave absorbing performance of the FeSiAl/MWCNT (2 wt.%) composite should be closely related to the special morphology of the flaky FeSiAl alloy and the synergetic effect between FeSiAl and MWCNT. As observed in Fig. 7(a,b), the deformation of the FeSiAl alloy leads to the great change of both the ε′ and ε′′ which represent the storage and dissipation abilities of electric energy, respectively. As the milling time increases, the ε′ values of the FeSiAl alloys vary in the range of 2.7–8.6, 6.6–9.1, and 4.5–5.7, respectively. The ε′′ curves display two obvious resonant peaks in 8–14 GHz and 15–18 GHz which correspond to two polarization relaxations. According to Debye theory^36^, the ε′′ consists of both polarization loss and conductive loss, described by the following equation,


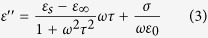


where σ is electrical conductivity, ω is angular frequency, τ is relaxation time, ε_s_ and ε_∞_ is static permittivity and relative permittivity, respectively. The polarization loss mainly originates from the interfacial polarization relaxations at the interfaces of FeSiAl-paraffin and FeSiAl-FeSiAl. Compared with irregular particles, the flaky ones possess higher aspect ratio and thus larger specific surface area, which contributes to an enhanced overall interfacial polarization loss. According to the equation 3 the conductivity loss is directly proportional to electrical conductivity. The severe deformation caused by milling could create a large number of structural defects in FeSiAl particles, which hinders the transfer of charges. Hence, the increased electrical resistivity of the particles may lead to the drop of permittivity. When the milling time extends to 16 h, the ε′′ considerably decreases relative to the raw alloy.

As a traditional magnetic material, the magnetic loss of the FeSiAl alloy plays a dominant role in tuning the microwave absorption performance. It can be seen from Fig. 7(c,d) that both the *μ*′ and *μ*″ of the flaky FeSiAl alloys are larger than those of the irregular one. Especially, the FeSiAl-8sample exhibits the largest *μ*′ and *μ*″ values of 1.58 and 0.54, respectively. The resonant peaks in the *μ*″ curves in low-frequency and high-frequency region can be assigned to the natural resonance and exchange resonance of the FeSiAl alloy, respectively. For irregular-shaped FeSiAl alloy, the permeability is generally restrained by Snoek’s limit^37^, whereas the FeSiAl alloy with flaky shape can overcome such limit. As observed in Fig. 7(b), the natural resonance frequency (*f*_*r*_) of the raw FeSiAl alloy is presumed to be below 2 GHz, while the *f*_*r*_ shifts to ~2.5 GHz when the alloy was milled. The relationship between permeability and resonance frequency of the irregular and flaky FeSiAl alloys follows the Equation 4 and 5, respectively.


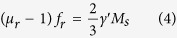



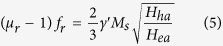


Where *μ*_r_, *f*_r_, *γ*′ and *M*_s_ are the initial permeability, resonance frequency, gyro-magnetic ratio and saturation magnetization, respectively. *H*_ea_ and *H*_ha_ represent the in-plane and out-of-plane anisotropy fields, respectively. Because *H*_ea_ ≪ *H*_ha_, the permeability of flaky particles would be higher than that of the irregular-shaped one, which is consistent with our results. Additionally, the exchange coupling reaction of the magnetic moment between particles can be enhanced with the increase of surface area^38^,^39^, which also benefits the increase of permeability of the milled FeSiAl alloys.

Among the raw and milled FeSiAl alloys, the relatively larger *ε*′′ and *μ*″ values of the FeSiAl-8 sample indicate the higher dielectric loss and magnetic loss, which contribute to the strongest microwave absorbing capability.

**Figure 8:**
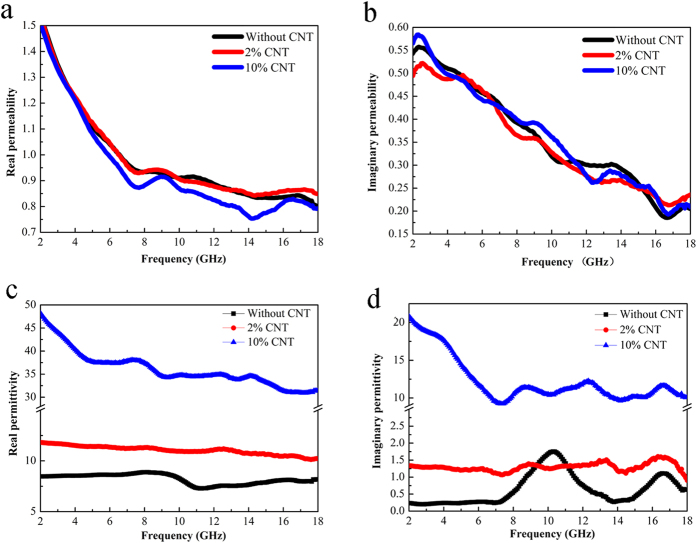
The frequency dependence of complexpermittivity and permeability of FeSiAl-8 and FeSiAl/MWCNT composites.

**Figure 9:**
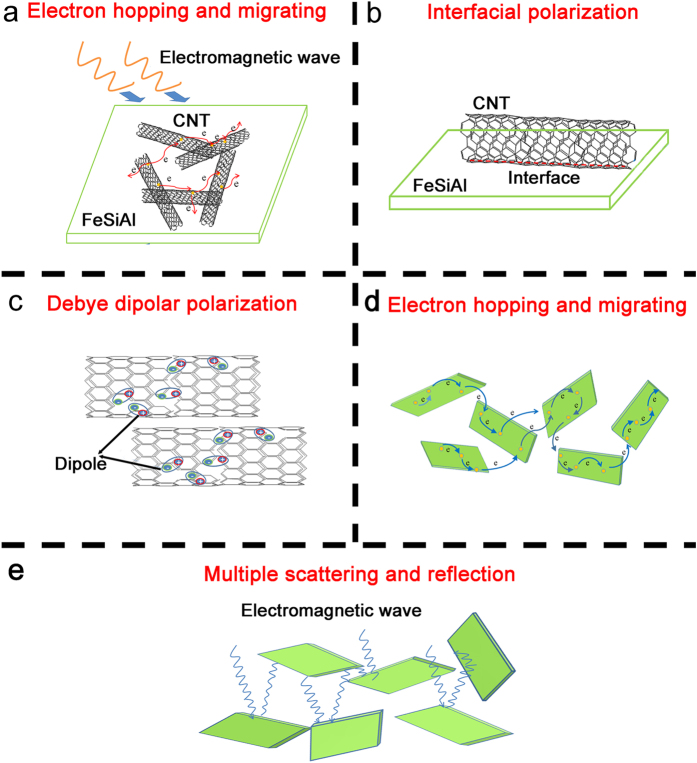
The illustration for main microwave attenuation mechanisms.

After mixing MWCNTs with FeSiAl-8 alloy, the further enhancement of the microwave absorbing performance is expected to originate from the synergetic effects of the MWCNTs and FeSiAl alloys. Because of the small addictive amount of MWCNTs in FeSiAl/MWCNT composites, the incorporation of MWCNTs has little effect on the permeabilities of the composites in terms of the measurement error, as illustrated in Fig. 8(a,b). However, the permittivities of the FeSiAl/MWCNT composites are apparently enhanced compared with pure FeSiAl-8 sample. Figure 8(c,d) shows the complex permittivities of the FeSiAl/MWCNT composites with different MWCNT amount. The *ε*′ and *ε*′′ of the FeSiAl-8 alloy fluctuate in the range of 6.5–8.1 and 0.2–1.7, respectively. After mixingwith MWCNTs with weight percentage of 2 wt.%, the *ε*′ value of the composite increases to 10.1–11.8 and the average *ε*′′ value increases to 1.3. As the MWCNTs weight percentage increases to 10 wt.%, the maximum values of *ε*′ and *ε*′′ considerably rise to 48.2 and 20.8 (at 2 GHz), respectively, which are several times larger than those of the other samples. Meanwhile, the *ε*′′ curves of the FeSiAl/MWCNT composites exhibit more resonant peaks compared with FeSiAl-8 sample. According to the abovementioned equation 3, the variation of permittivity caused by the addition of MWCNTs can be explained as follows. Firstly, the addition of MWCNTs increases the conductivity of the composite and provides additional conductive paths for electron hopping and migrating as shown in Fig. 9(a), which enhances the conductive loss. Secondly, the addition of MWCNTs introduces more polarization relaxations which are evidenced by the increase in the number of resonant peaks in ε′′ curves. The Debye dipolar polarization relaxation can be evaluated by the Cole-Cole plot as expressed by^40^,^41^,


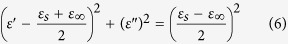


**Figure 10:**
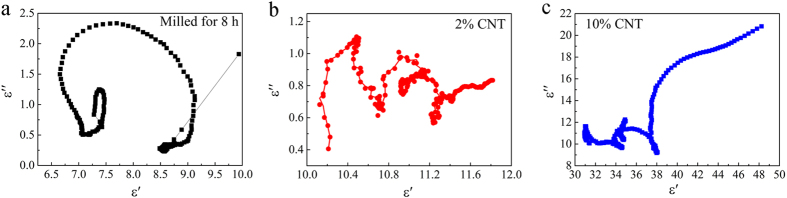
The *ε*′′–*ε*′ curves of (**a**) FeSiAl-8, (**b**) FeSiAl/MWCNT composite (2 wt.% MWCNT), (**c**) FeSiAl/MWCNT composite (10 wt.% MWCNT).

Thus, the plot of *ε*′′ versus *ε*′ would be a single semicircle representing one Debye relaxation process. As shown in Fig. 10(a), two obvious Cole–Cole semicircles are found in raw FeSiAl-8 alloy, suggesting the presence of two Debye relaxation processes. After mixing with MWCNTs, several semicircles are clearly observed for FeSiAl/MWCNT composites in Fig. 10(b,c)^42^. The multiple Debye relaxation processes include the dipole relaxations associated with the surface functional groups and defects in MWCNTs (Fig. 9(c))^43^,^44^, and the dipole relaxations at interfaces i.e. FeSiAl-FeSiAl, MWCNT-FeSiAl, FeSiAl-paraffin and MWCNT-paraffin interfaces. The increase of dipole polarization relaxation in FeSiAl/MWCNT composites greatly increases the polarization loss. In summary, the enhanced conductivity loss and polarization loss account for the increase of *ε*′′ of the FeSiAl/MWCNTs composites.

Compared with FeSiAl-8 sample, the larger *ε*′′ of the FeSiAl/MWCNTs composite suggests enhanced overall losses (magnetic loss+ dielectric loss) which should induce stronger microwave attenuation capability. However, as observed in Fig. 6(b), only the FeSiAl/MWCNTs composite with moderate amount of MWCNTs (2 wt.%) displays enhanced microwave absorbing property while the composite with excessive addition of MWCNTs (10 wt.%) possesses much poorer absorbing capability. To explain this phenomenon, impedance matching which is another key factor for determining the microwave absorbing property should be taken into account. It is known that good impedance matching which requires the balance between permittivity and permeability is the prerequisite for EM wave entering absorber. The impedance matching degree ∆ of the FeSiAl-8 and FeSiAl/MWCNTs can be calculated in the following equations^45^,^46^:


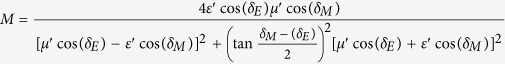



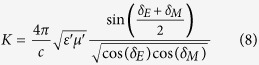



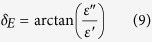



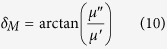


**Figure 11:**
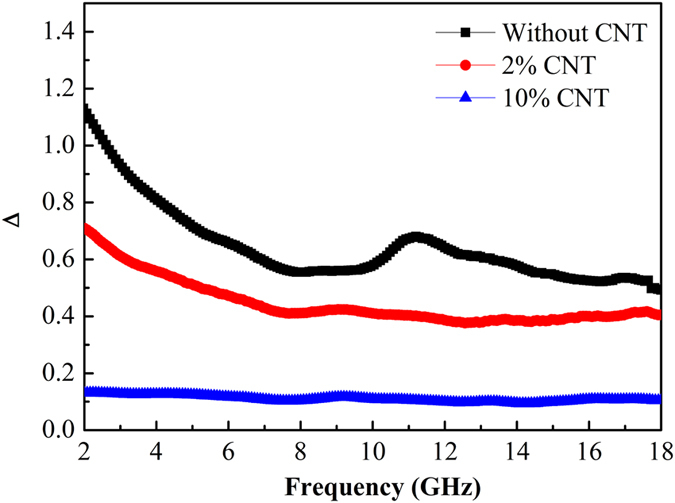
The impedance matching degree (∆) curves of FeSiAl-8 and FeSiAl/MWCNT composites.

Figure 11 depicts the impedance matching degree of FeSiAl-8 alloy and FeSiAl/MWCNTs composites at a layer thickness of 2 mm. The value of ∆ for the FeSiAl/MWCNTs (10 wt.%) composite was far beyond 1.0, implying the poor impedance matching. In this case, the strong reflection of EM wave on the absorber surface would restrict the absorption of electromagnetic wave inside the absorber.

From the above analysis, we can conclude that the good impedance matching and the strong EM wave attenuation capability contribute to the excellent microwave absorbing performance of the FeSiAl/MWCNT (2 wt.%) composite. Firstly, the balance between permittivity and permeability of the composite can ensure the incident waves enter the absorber to the greatest extent. Secondly, the strong magnetic loss and the enhanced dielectric loss contribute to the strong microwave attenuation (as illustrated in Fig. 9(b,c)). Thirdly, the established 3D conductive paths by MWCNTs and FeSiAl sheets for electron hopping and migrating could transform the incident wave into heat or other forms of energy (as illustrated in Fig. 9(a,d))^43^. In addition, the thin FeSiAl sheets with large aspect ratio can increase the propagation paths for the incident waves inside the sample by multiple scattering and reflection, as illustrated in Fig. 9(e).

## Conclusions

A serial of FeSiAl alloy/MWCNT composites were prepared by ball milling method with tunable morphology, aspect ratio of FeSiAl alloy and various content of MWCNTs in the composite. The magnetic loss of the composite strongly depends on the morphology and structure characteristics of the FeSiAl component. With optimized ball milling time, the composite displays high magnetic loss value due to the increased anisotropy field. Moreover, the dielectric loss of the composite was improved by tuning the addition amount of MWCNTs due to the enhancement of interfacial polarizations, dipolar polarizations and the increase of conductive paths. After ball milling for 8 h, a minimum RL value of −42.8 dB at 12.3 GHz was achieved in FeSiAl/MWCNT composite with 2 wt.% MWCNT content at a very thin coating thickness of 1.9 mm. The outstanding microwave absorption properties as well as the facile fabrication method endow the FeSiAl/MWCNT composite promising applications in both military and civil fields.

## Additional Information

**How to cite this article**: Huang, L. *et al.* Flaky FeSiAl alloy-carbon nanotube composite with tunable electromagnetic properties for microwave absorption. *Sci. Rep.* **6**, 35377; doi: 10.1038/srep35377 (2016).

## Figures and Tables

**Table 1:** The *M*_*s*_, *M*_*r*_ and *H*_*c*_ values of the raw and as-milled FeSiAl alloys.

Sample	*M*_*s*_(emu/g)	*M*_*r*_(emu/g)	*H*_*c*_(Oe)
Raw	130.11	0.08	1.00
Milled for 4 h	134.98	1.31	12.59
Milled for 8 h	127.03	1.51	18.19
Milled for 16 h	126.60	1.84	18.44

**Table 2:** Microwave absorption properties of FeSiAl-based absorbers reported in this work and recent literatures.

Filler	Matrix	Loading (wt%)	Thickness (mm)	Effective bandwidth (GHz)	Minimum RL (dB)	Ref
FeSiAl/MWCNT	silicone rubber	50	1	—	−7.3 (at 2.62 GHz)	23
FeSi	paraffin	75	2	0.4	−13.1 (at 2.9 GHz)	24
FeSiAl	paraffin	35	4	3.2	−39.67 (at 1.4 GHz)	25
FeSiAl/BaTiO_3_	epoxy	4	4	2.8	−18.1 (at 3.1 GHz)	26
Fe-Ti–Si-Al	epoxy	98	3	1.24	−23.1 (at 1.55 GHz)	27
FeSiAl/graphite	paraffin	40	3	2	−21 (at 6.7 GHz)	28
FeSiAl	paraffin	35	5	1.2	−13.4 (at 1.44 GHz)	21
FeSiAl/MWCNT	paraffin	40	1.9	3.9	−42.8 (at 12.3 GHz)	this work
